# Effects of Ageing on Donkey Meat Chemical Composition, Fatty Acid Profile and Volatile Compounds

**DOI:** 10.3390/foods11060821

**Published:** 2022-03-12

**Authors:** Paolo Polidori, Giuseppe Santini, Yulia Klimanova, Jing-Jing Zhang, Silvia Vincenzetti

**Affiliations:** 1 School of Pharmacy, University of Camerino, Via Gentile da Varano, 62032 Camerino, MC, Italy; 2 School of Biosciences and Veterinary Medicine, University of Camerino, Via Circonvallazione 93, 62024 Matelica, MC, Italy; giuseppe.santini@unicam.it (G.S.); yulia.klimanova@unicam.it (Y.K.); jingjing.zhang@unicam.it (J.-J.Z.); silvia.vincenzetti@unicam.it (S.V.)

**Keywords:** donkey meat, meat quality, meat flavour, volatile compounds, fatty acid profile

## Abstract

Donkey meat samples obtained from muscle *Longissimus Thoracis Lumborum* (LTL) taken from 14 entire donkey males slaughtered at 20 months and aged for 1, 8 and 15 days were analysed with the aim of determining the chemical composition, physical attributes, fatty acid profile and volatile compounds. Ageing did not significantly affect the chemical composition and colour parameters, while cooking loss was significantly (*p* < 0.05) higher at 8 and 15 days of ageing. Thiobarbituric acid reactive substances (TBARS) content significantly (*p* < 0.01) increased during ageing, while shear force values significantly (*p* < 0.01) decreased. Ageing significantly (*p* < 0.05) increased polyunsaturated fatty acids (PUFAs) determined both at 8 and 15 days after slaughter. Volatile compounds were analysed using solid-phase microextraction (SPME) and gas chromatography–mass spectrometry (GC–MS). Among 109 volatile compounds determined in donkey meat, hydrocarbons were the most common molecules detected. Ageing affected 21 of the detected volatile compounds; both total aldehydes and total ketones contents were significantly (*p* < 0.05) higher 15 days after slaughter. Total furans and total alcohols were significantly (*p* < 0.01) higher 15 days after slaughter, as well. Significant modifications of donkey meat volatile compounds can be attributed to ageing periods longer than 7 days.

## 1. Introduction

Flavour is a very important sensorial characteristic in determining meat total quality, and for this reason, several studies have been performed in order to understand the chemistry of meat flavour and to determine the different factors that can influence flavour formation [1]. In previous studies, over 1000 volatile compounds have been determined in different kinds of meat; raw meat can be considered as a source of non-volatile precursors of volatile compounds, namely free amino acids, peptides, saccharides, inorganic salts, and inorganic acids [2]. Ageing represents an important process, during which muscle is transformed into meat [3]. Reactions that take place during this process influence meat quality, especially meat flavour. In fact, ageing improves the eating quality and the release of compounds such as free amino acids or fatty acids, which are the substrates for the formation of flavour compounds [4]. Another important fraction in the formation of volatile compounds is represented by fat content, the degradation of which causes the formation of several volatile compounds, such as carboxylic acids, alcohols, esters, aliphatic hydrocarbons, aldehydes or ketones [5].

Equid meat shows interesting quality parameters, such as low fat and cholesterol content, and represents a good source of iron [6,7]. Equid meat also presents a high ratio of unsaturated fatty acids compared to saturated fatty acids, with good content of essential fatty acids [8,9]. Because of recent scientific findings, horse meat consumption is slowly increasing in several European countries where it is considered a good red meat alternative [10]. Donkey meat represents a very small niche in equid meat consumption, even if its chemical composition and its physical characteristics are very similar to those of horse meat [11]. Use of local donkey breeds for meat production is important for promoting and protecting animal biodiversity, which can also contribute to the valorisation of local production systems, due to the high adaptation of donkeys and their resistance to diseases [12].

Meat volatile compounds are influenced by the animal’s genetics, slaughter age and diet [13,14]. Previous studies have correlated specific volatile compounds with animal diet [15,16]. Volatile compounds such as terpenoids, phenols and the diketone 2,3-octanedione were associated with diets based on pasture [17]. On the other hand, lactones, branched fatty acids and compounds such as 2,3-butanedione and furan, 2-pentyl were associated with diets based on cereals [18]. 

The aim of the present study was to evaluate the effects of the ageing period (1, 8 and 15 days) on the chemical composition, physical attributes, fatty acid profile and volatile organic compounds in previously vacuum-packaged, cooked meat obtained from 14 crossbred donkey males reared in the Apennine mountain region of Italy.

## 2. Materials and Methods

### 2.1. Animals and Diets

This study was performed during winter/spring 2021 and was approved by the Animal Welfare Committee of the University of Camerino (Ref. 7/2021). Fourteen crossbred (Martina Franca × Amiata) entirely male donkey foals were obtained from the farm named “Asinomania”, located in Introdacqua, Aquila Province, Abruzzo Region, Italy. Foals were reared with their mothers on pasture, naturally suckling colostrum at birth and later milk from their dams until weaning at the age of 7 months. After weaning, foals were fed with a commercial feed, and the amount was gradually increased, starting with small quantities to reach the final amount of 1.5 kg of fodder/foal-day. The commercial feed chemical composition showed: crude protein (16.3%), crude fibre (7.6%), ash (5.7%), fat (4.0%) and sodium (0.3%). The ingredients in the commercial concentrate feed were, in unknown quantities, barley, corn, soybean flour, wheat germ, alfalfa, sugar cane molasses, dry beet pulp, animal fat, calcium carbonate, sodium chloride, powder lactose and a vitamin and mineral mix.

### 2.2. Slaughter Procedure and Sampling

The 14 donkeys were slaughtered at the age of 20 months. According to European Community laws on Animal Welfare (1/2005EC), they were transported the day before slaughter to the European Community-approved abattoir, then slaughtered and dressed according to current European Union regulations (1099/2009EC). Transport to the slaughter house took about 90 min. The animals were fasted (only water was available) for 12 h before slaughter; a conventional captive bolt was used for stunning, then the carotid arteries and jugular veins were severed, resulting in fast animal bleeding, causing heart failure and death. Carcasses were skinned and eviscerated; all the non-carcass components (skin, head, feet, heart, lungs, liver, spleen, and the entire digestive tract) were removed. Carcasses were then transferred to a cold room at a temperature of 3 °C and stored suspended by the hind legs. The day after slaughter, samples of Longissimus Thoracis et Lumborum (LTL) muscle (about 400 g of each sample) were removed from the right-hand side of each carcass, between the fourth and ninth rib, and transported in refrigerated bags to the lab, where each sample was divided into four pieces, vacuum-packaged, stored at 3 °C, and then randomly assigned to one of the different ageing periods (1, 8, and 15 days). The pH was measured at 1, 8, and 15 days after slaughter by inserting a pH-meter glass probe (Portamess Knick mod.910) into the sample 2.0 cm below the surface [19]. 

### 2.3. Chemical Analysis

Chemical composition (moisture, protein, intramuscular fat, ash) and physical attributes were determined in triplicate [20]. All samples were vacuum-packaged using LARI/3 pn-VG packaging machine (CAVECO, Milano, Italy), then all vacuum bags were stored at 3 °C and were rotated every 24 h to reduce the effects of light intensity differences and possible temperature variations on meat surface [21]. Ten samples were obtained and analysed at each ageing time (1, 8, and 15 days) for physical-chemical analysis. Fatty acid composition was determined after lipid extraction [22]; later, fatty acid methyl esters [23] were determined using a gas chromatograph (model CP 9003, Agilent Technologies, Santa Clara, CA, USA) with a flame ionization detector and a fused-silica capillary column and a film thickness of 0.2 μm, packed with CP Sil 88 (50 m × 0.25 mm i.d.). Fatty acid identification was performed by comparing the retention times with those of known fatty acids, and the results were expressed as mg/100 g of fat. Atherogenic index (AI) and thrombogenic index (TI) were calculated using the following equations, in order to evaluate the atherogenic activity of C12:0, C14:0, and C16:0 and the thrombogenic activity of C14:0, C16:0, and C18:0 [24]:

AI: C12:0 + (4 × C14:0) + C16:0 (MUFA + PUFA)

TI: (C14:0 + C16:0 + C18:0) (0.5 × MUFA) + O.5 × PUFA n − 6 + 3 × PUFA n − 3 + (n − 3/n − 6)

### 2.4. Water-Holding Capacity and TBARS Measurement

The water-holding capacity (WHC) was measured using two methods, evaluating drip loss (DL) and cooking loss (CL) [25]. To determine drip loss, a sample of donkey LTL muscle weighing approximately 80–100 g and of 1.5 cm in thickness was weighed and put on top of a net, inside a container that was closed after filling in order to avoid evaporation into the environment. This container was placed in a refrigerated chamber at 4 °C for 48 h, and after this period, was weighed again. The percentage of drip loss water was calculated as [26]:

DL = (initial fresh meat weight − meat after 48 h weight) × 100 (initial fresh meat weight)

For CL determination, LTL samples were roasted for 15 min at 200 °C on a metal tray using an electric oven; heating treatment was considered complete when the internal temperature in all of the samples reached 73 °C, monitored with thermocouples. For determining DL, 0.3 g was collected from each sample and then centrifuged at 30,000× *g* for 1 h; then, the samples were dried and weighed again, and the centrifugation loss was calculated as the difference in weight before and after centrifugation [27]. CL was measured by evaluating the difference in weight between the cooked and raw samples [27]:

CL = (weight loss/initial fresh meat weight) × 100

Determination of thiobarbituric acid reactive substances (TBARS) was performed on donkey meat LTL samples 1, 8 and 15 days after slaughtering, according to the extraction method described by Dominguez et al. (2014). LTL sample (2 g) was dispersed in 5% trichloroacetic acid (10 mL) and homogenized in an Ultra-Turrax (Ika T25 basic, Staufen, Germany) for 2 min. The homogenate was maintained at −10 °C for 10 min and centrifuged at 2050× *g* for 10 min. The supernatant was filtered through a Whatman No. 1 filter paper. The filtrate (5 mL) was reacted with 0.02 M TBA solution (5 mL) and incubated in a water bath at 96 °C for 40 min. The absorbance was measured at 532 nm; TBARS values were calculated from a standard curve of malonaldehyde with 1,1,3,3-tetramethoxypropane, and the concentration of lipid oxidation was expressed as milligrams of malondialdehyde (MDA)/kg sample [28]. 

### 2.5. Physical Analysis

Shear force value was determined on LTL muscles 1, 8 and 15 days after slaughter; chops (each 2.5 cm thick) were roasted at an oven temperature of 200 °C, to an internal temperature of 73 °C monitored with thermocouples [29]. From each sample, 8 cores (1.2 cm in diameter) were sheared with a Warner–Bratzler operating head mounted on an Instron apparatus 4411 (Instron, High Wycombe, UK). Peak or maximum shear force was expressed in N/cm^2^.

Muscle colour parameters were measured 1, 8, and 15 days after 1 h of oxygen exposure at room temperature using a Minolta CM-3600 D spectrophotometer (Konica Minolta Holdings Inc., Marunouchi, Chiyoda, Tokyo, Japan), with a measured area diameter of 8 mm, a standard illuminant D65, and an observer angle of 10°. Black and white calibrations were made with the standard tiles in order to determine the L* (lightness), a* (redness), and b* (yellowness) [30]. After placing the measuring lens on the meat surface, it was turned through 0°, 45°, and 90° (clockwise) to obtain three different reflectance measurements that were later averaged.

### 2.6. Volatile Analysis

The same cooking procedure as described for WHC determination was applied for LTL samples belonging to the same ageing time (1, 8, and 15 days) used for volatile organic compound determination. An SPME device (Supelco, Bellefonte, PA, USA) containing a fused silica fibre (10 mm length) coated with a 50/30 μm layer of DVD/CAR/PDMS was used. The vial was left at 35 °C in a thermo block for 15 min to equilibrate its headspace. Then, an SPME fibre was exposed to the headspace while maintaining the sample at 35 °C for 30 min. The compounds absorbed by the fibres were identified and quantified by gas chromatographic analysis using MS detectors.

A gas chromatograph equipped with mass selective detector (GC-MSD) (Agilent, Santa Clara, CA; Agilent 6890N with Agilent 5973N) was used. The compounds were separated using a DB-624 capillary column (J&W scientific: 30 m × 0.25 mm id, 1.4 μm film thickness (Folsom, CA, USA). The SPME fibre was desorbed and maintained in the injection port at 260 °C for 5.0 min. The sample was injected in splitless mode. Helium was used as a carrier gas with a linear velocity of 40 cm/s. The temperature programme was isothermal for 10 min at 40 °C, raised to 200 °C at 5 °C min−1, and then raised to 250 °C at 20 °C min^−1^, and held for 5 min; total run time was 49.5 min [31]. The injector and detector temperatures were both set at 260 °C. The mass spectra were obtained using a mass selective detector working in electronic impact at 70 eV, with a multiplier voltage of 1953 V and collecting data at 6.34 scans/s over the range *m*/*z* 40–300. Compounds were identified by comparing their mass spectra with those contained in the National Institute of Standards and Technology, Gaithersburg (NIST08) library in accordance with the standards of the International Organization of the Flavor Industry (IOFI, http://www.iofi.org/ accessed on 30 September 2021) statement.

The results from the HS-SPMEGC-MS analysis of volatile components extracted by the SPME fibre were expressed as percentages, obtained by dividing the area of each component by the total area of all isolated components. Data were analysed using MSD Chem Station software (Version 4.03.016, Agilent, Santa Clara, CA, USA). Results from volatile analyses were shown in total area counts.

### 2.7. Statistical Analysis

Results were submitted to a one-way analysis of variance, and the differences between ageing periods were evaluated using Tukey’s test [32]. Data were expressed as least squares mean and mean standard error; statistical significance was set as *p* < 0.05.

## 3. Results and Discussion

Donkey meat chemical and physical parameters are shown in Table 1. Meat chemical composition was not significantly affected by ageing period, as previously determined also in Martina Franca male foals slaughtered at 18 months of age [33]. The WHC was measured using CL and DL (Table 1); CL was significantly (*p* < 0.05) influenced by the ageing period; results obtained were in the range of 33–37% previously determined in other studies [31,34]. DL remained basically unchanged during different ageing periods; CL was mainly due to the loss of water, showing a major decrease after cooking, while meat fat and protein contents slightly increased; a high loss of water implies that meat is perceived as less juicy [34]. Moreover, CL increases about 20–30% when cooking temperature increases from 70 °C to 80 °C [35]; small differences in the cooking temperature can greatly affect the result. It is not easy to compare with other data obtained using different temperatures [25].

Ageing significantly (*p* < 0.01) increased TBARS content (Table 1); values determined in this study were similar to those obtained in fresh foal meat [36]. The ageing process did not significantly affect meat pH values (Table 1), close to 5.6 in all determinations, as noticed also in horse meat obtained from an Italian autochthonous local breed named Catria [37]. Values of pH measured in donkey meat at 2, 8 and 15 days after slaughter confirmed that a normal acidification was achieved during post-slaughter metabolism; the rate of pH decline stopped 24 h after slaughtering, showing from that moment the typical range of 5.4–5.7 in values obtained in several previous studies [38]. The TBARS values determined in this study were similar to the results obtained from Galician Mountain foal meat aged for 4, 7, 11 and 14 days; lipid stability significantly decreased during ageing [28]. In several studies, it has been reported that protein oxidation is partially linked to lipid oxidation as measured by TBARS [39]. A previous study based on beef [40] showed relatively little change during the ageing period of 0–2 weeks, whereas great variations were observed for the ageing period of 2–4 weeks. 

Ageing period did not affect colour parameters; these results were consistent with those reported in a review showing data concerning horse meat [41]. Shear force values significantly (*p* < 0.01) decreased both after 8 and 15 days of ageing, confirming results obtained from evaluations of donkey meat samples after 4 and 7 days of ageing [11]. 

The fatty acid contents of donkey muscle are shown in Table 2. The two most represented fatty acids in all three different ageing periods were oleic acid (18:1cis9) and palmitic acid (C16:0), confirming results previously determined in meat obtained from Martina Franca donkey males [42]. Saturated fatty acid (SFA) and monounsaturated fatty acid (MUFA) contents were similar at 1, 8 and 15 days after slaughter (Table 2); a significantly (*p* < 0.05) higher content of polyunsaturated fatty acids (PUFA) was found at 8 and at 15 days after slaughter, mainly due to a significant (*p* < 0.05) increase in n − 3 essential fatty acids. The PUFA/SFA ratio was, respectively, 0.65, 0.71 and 0.67 at days 1, 8 and 15 after slaughter (Table 2). Donkey meat shows high nutritional value, due to its significantly (*p* < 0.05) higher PUFA content after 8 and 15 days of ageing, some of which plays key roles as precursors of antithrombotic factors [43]. The ratio of PUFA/SFA and the n − 6/n − 3 ratio influence the nutritional value of lipids in red meats; fats containing a lower SFA content and higher PUFA content are considered as ideal [44]. The PUFA/SFA as well as the n − 6/n − 3 fatty acid ratios are primary values used to measure the nutritional quality of foods for human nutrition [45]. The donkey meat fatty acid profile determined in this study confirmed that equid meat is very close to the recommended benchmark value for the PUFA:SFA (>0.7) and the n − 6/n − 3 ratio (<5), compared to the values determined in beef, lamb and pork meat [46].

Fat quality is more important than the total fat content in the quantification of cardiovascular disease risk; for this reason, the atherogenicity index (AI) and the thrombogenicity index (TI) are usually considered very important nutritional parameters [47]. Results obtained in this study were similar to those obtained in meat produced both by donkey males and mules [48]. Data obtained in this study confirmed the healthy fatty acid profile previously determined for horse meat obtained from rearing Sanfratellano and Halflinger foals [49].

Ageing effects on volatile compounds of donkey meat aged for 1, 8, and 15 days after slaughter are shown in Table 3. A total of 109 volatile compounds were identified in cooked donkey meat samples during ageing; they were classified according to their chemical families, namely 46 hydrocarbons, 6 aromatic hydrocarbons, 16 aldehydes, 13 ketones, 6 furans, 12 alcohols, 3 carboxylic acids, 5 nitrogen compounds, and 2 sulphur compounds. After 15 days of ageing, propanal, butanal, 3-methyl, pentanal, heptanal, and benzeneacetaldeyde showed significantly (*p* < 0.05) higher content, while nonanal content increased significantly (*p* < 0.01) as well. Total aldehyde content was significantly (*p* < 0.05) higher after 15 days. Furan, 2-ethyl-, furan, 2-pentyl-, 2-n-Butyl furan, and total furans at day 8 showed significantly higher values (*p* < 0.05). After 15 days of ageing, Furan, 2-ethyl-, 2-n-Butyl furan significantly (*p* < 0.05) increased, while Furan, 2-pentyl- and total furans showed significantly (*p* < 0.01) higher values compared to day 2 and day 8. Among 109 volatile compounds detected in donkey meat, only 21 compounds, namely 1 hydrocarbon, 3 aromatic hydrocarbons, 6 aldehydes, 4 ketones, 3 alcohols, 1 carboxylic acid and 3 nitrogen compounds, were significantly affected by aging time. The lack of significant differences in volatile compound contents during ageing periods has been attributed to high variability in the raw data and interactions during the formation of the volatile compounds [50,51]. 

The effects of ageing period on hydrocarbon and aromatic hydrocarbon compounds detected in donkey meat samples are shown in Table 4. Among hydrocarbons, pentane content was significantly (*p <* 0.05) increased after 15 days of ageing; total hydrocarbon content increased both after 8 and 15 days of ageing, but not significantly. Among aromatic hydrocarbons, benzene, ethylbenzene and 3-carene were affected by ageing period, showing significant (*p* < 0.05) increases in their content after both 8 and 15 days. Total aromatic hydrocarbon content was significantly (*p* < 0.05) higher after both 8 and 15 days of ageing. 

Effects of ageing on ketone and alcohol content in donkey meat after 2, 8 and 15 days of ageing are shown in Table 5. Total ketones were significantly (*p* < 0.05) higher after 15 days of ageing. Among 13 ketones detected in donkey meat, 2-Heptanone showed the highest content, followed by 2,3-Butanedione. Four out of 13 detected ketones showed a significant (*p* < 0.05) increase after 15 days of ageing (Table 5). Total alcohol content was significantly (*p* < 0.01) higher after 15 days of ageing. 1-Hexanol and 1-Pentanol were the two most represented alcohols among a total of 12 compounds belonging to this category and detected in donkey meat (Table 5).

The effects of ageing on carboxylic acids, total nitrogen compounds, and total sulphur compounds are shown in Table 6. Total carboxylic acid content in donkey meat was significantly (*p* < 0.05) increased at both 8 and 15 days after slaughtering; in this category, butanoic acid was the most represented, at both 8 and 15 days after slaughter. Total nitrogen compounds significantly (*p* < 0.05) decreased at 8 and 15 days after slaughter. Total sulphur compounds were not affected by ageing period (Table 6).

Raw donkey meat is a rich reservoir of precursor molecules of volatile compounds. Total volatile compounds detected in this study for donkey meat were basically the same as determined in previous studies performed on horse meat [52]. Furans and aldehydes are very important for odour, as determined in previous studies [53]. Furans are normally associated with heat, and they are generated from amino acids [54]; the significant increases in some furans during ageing time could be explained by the increase in free amino acids during the storage time and high cooking temperatures, which lead to the Maillard reaction. 

Hydrocarbons in the volatile compound category were the most represented in donkey meat samples, but only one (pentane) out of 46 total compounds was significantly affected by ageing period, confirming results previously determined in horse meat [55]. The authors attributed this result to the late development of hydrocarbons during meat ageing, normally not before 15 days. This lack of significant differences among different ageing periods could also be related to the low intramuscular fat content in donkey meat, given that fat is the main source responsible for meat flavour and odour development [56].

Generally, the results obtained in the present paper are consistent with data reported in previous studies based on the evaluation of the increase in volatile compounds during ageing of beef and lamb [57]. The increase in volatile compounds during the first week of vacuum aging can be linked to the chemical modifications of fat and protein molecules during storage [58].

## 4. Conclusions

The results obtained in this study showed that the ageing process affects some important donkey meat quality parameters. Donkey meat tenderness, PUFA content and volatile profile were significantly affected by the ageing period. Further studies are necessary to evaluate the effects of different production systems on donkey carcass and meat quality parameters, with the aim of enhancing local donkey breeds, preserving animal biodiversity and creating opportunities for characterizing a niche product, such as donkey meat, that could represent a good strategy for local farmers living in marginal areas, especially in the highlands of central and southern Italy.

## Figures and Tables

**Table 1:** Chemical and physical donkey meat parameters determined at different ageing periods.

KERRYPNX		Ageing		Significance	SEM
	1 d	8 d	15 d		
Moisture (%)	73.28	73.47	73.88	n.s.	0.91
Protein (%)	22.14	21.91	21.80	n.s.	0.57
Fat (%)	3.12	3.15	2.97	n.s.	0.49
Ash (%)	1.45	1.47	1.38	n.s.	0.28
Drip Loss (%)	3.96	3.89	3.94	n.s.	0.51
Cooking loss (%)	30.7 ^a^	36.2 ^b^	35.9 ^b^	*	0.88
TBARS values (mg MDA/kg muscle)	0.33 ^A^	1.24 ^B^	1.32 ^B^	**	0.08
pH value	5.66	5.67	5.58	n.s.	0.03
Lightness L	39.4	40.1	41.13	n.s.	0.40
Redness a	11.91	10.87	11.45	n.s.	0.55
Yellowness b	8.28	8.46	8.32	n.s.	0.44
Warner Bratzler Shear Force (N/cm^2^)	61.6 ^A^	55.8 ^B^	53.9 ^B^	*	0.19

Significance: * (*p* < 0.05); ** (*p* <0.01). n.s.: not significant. Different letters in the same row denote statistical difference: (^a,b^ *p* < 0.05; ^A,B^ *p* < 0.01).

**Table 2:** Fatty acid composition (% total fatty acids) determined in donkey muscle. LTL aged for 1, 8, and 15 days.

Fatty Acid	1 Day	8 Days	15 Days	SEM	*p*-Value
C12:0	0.26	0.21	0.24	0.005	n.s
C14:0	3.88	3.24	3.63	0.002	n.s
C15:0	0.47	0.39	0.44	0.008	n.s
C16:0	27.8	28.1	28.0	0.38	n.s
C16:1	3.63	4.00	3.81	0.22	n.s
C17:0	0.58	0.47	0.52	0.005	n.s
C18:0	7.99	6.81	7.60	0.24	n.s
C18:1n−9	28.7	29.0	28.4	0.482	n.s
C18:2n − 6	20.3	20.9	20.2	0.202	n.s
C18:3n − 3	3.17	4.20	3.60	0.11	n.s
C20:1n−9	0.32	0.34	0.33	0.007	n.s
C20:2n − 6	0.23	0.19	0.30	0.008	n.s
C20:3n − 6	0.28	0.17	0.19	0.005	n.s
C20:4n − 6	2.27	2.10	2.43	0.12	n.s
C20:5n − 3	0.19	0.24	0.32	0.03	n.s
SFA	40.98	39.22	40.43	0.421	n.s
MUFA	32.65	33.34	32.54	0.356	n.s
PUFA	26.44 ^a^	27.80 ^b^	27.04 ^b^	0.267	*
PUFA/SFA	0.65	0.71	0.67	0.006	n.s
Σn − 3	3.36 ^a^	4.44 ^b^	3.92 ^b^	0.014	*
Σn − 6	23.08	23.36	23.12	0.221	n.s
Σn − 6/Σn − 3	6.87	5.26	5.90	0.035	n.s
AI	0.74	0.68	0.72	0.019	n.s
TI	1.04	0.91	0.99	0.025	n.s

Significance: * (*p* < 0.05). n.s.: not significant. Different letters in the same row denote statistical difference: (^a,b^ *p* < 0.05).

**Table 3:** Effects of ageing period on donkey meat aldehyde and furan content (AU × 10^3^/g).

Volatile Compound	1 Day	8 Days	15 Days	SEM	*p*-Value
Propanal	354.3 ^a^	349.6 ^a^	462.5 ^b^	122.7	*
Propanal, 2-methyl-	71.5	76.9	71.7	11.2	n.s.
Butanal	38.7	32.8	47.8	2.77	n.s.
Butanal, 3-methyl-	64.8 ^a^	88.1 ^a^	173.8 ^b^	41.1	*
Butanal, 2-methyl-	209.4	355.2	256.4	72.2	n.s.
Pentanal	994.9 ^a^	1241.7 ^b^	1564.2 ^b^	269.8	*
Hexanal	17854.1	16815.5	18546.6	3139.2	n.s.
Furfural	16.2	20.5	21.6	1.19	n.s.
2-Hexenal, (E)-	15.7	34.2	37.3	1.96	n.s.
Heptanal	369.5 ^a^	328.2 ^a^	583.8 ^b^	142.8	*
Benzaldehyde	121.3	153.8	187.4	24.2	n.s.
Benzeneacetaldeyde	34.7 ^a^	32.9 ^a^	88.7 ^b^	3.68	*
2-Octenal, (E)-	20.3	23.1	21.6	1.48	n.s.
Nonanal	290.8 ^A^	278.2 ^A^	595.2 ^B^	71.8	**
Benzaldehyde, 3-ethyl-	36.5	41.7	42.4	4.57	n.s.
2,4-Decadienal	17.2	15.4	16.8	0.53	n.s.
**Total aldehydes**	**20,509.9 ^a^**	**19,887.8 ^a^**	**22,717.8 ^b^**	**3918.2**	*
Furan, 3-methyl-	25.4	24.7	27.9	2.92	n.s.
Furan, 2,5-dihydro	23.6	22.8	25.7	1.47	n.s.
Furan, 2-ethyl	83.7 ^a^	97.6 ^a^	287.5 ^b^	37.4	*
2-n-Butyl furan	74.2 ^a^	82.3 ^a^	174.7 ^b^	17.3	*
Furan, 2,3-dihydro-3-methyl	21.3	22.2	27.3	9.4	n.s.
Furan, 2-pentyl-	579.4 ^A^	538.5 ^A^	1533.6 ^B^	371.2	**
**Total furans**	**807.6 ^A^**	**788.1 ^A^**	**2096.7 ^B^**	**442.3**	**

Significance: * (*p* < 0.05); ** (*p* < 0.01). n.s.: not significant. Different letters in the same row denote statistical difference: (^a,b^ *p* < 0.05; ^A^^,^^B^ *p* < 0.01).

**Table 4:** Effects of ageing period on donkey meat hydrocarbon and aromatic hydrocarbon content (AU × 10^3^/g).

Volatile Compound	1 Day	8 Days	15 Days	SEM	*p*-Value
Pentane	22.0 ^a^	23.4 ^a^	38.5 ^b^	2.47	*
n-Hexane	571.3	599.3	485.7	99.5	n.s.
Hexane, 2,2-dimethyl-	185.8	196.7	174.1	27.2	n.s.
Isopropylcyclobutane	24.8	28.1	27.8	2.11	n.s.
Heptane	49.4	45.2	46.4	5.72	n.s.
Pentane, 2,3,4-trimethyl	64.9	67.3	64.2	6.98	n.s.
Heptane, 3,3,4-trimethyl	85.1	81.5	85.6	9.20	n.s.
Hexane, 2,2,4-trimethyl-	116.2	120.5	121.6	11.9	n.s.
2-Heptene, 3-methyl-	115.7	134.2	137.3	19.6	n.s.
Octane	169.5	132.2	183.8	14.8	n.s.
2-Octene, (E)	11.3	13.8	11.8	0.24	n.s.
Octane, 2-methyl-	14.7	12.8	18.7	1.88	n.s.
Heptane, 2,3-dimethyl	40.3	43.1	41.6	1.48	n.s.
4-Octene, (E) -dimethyl-	10.8	12.2	12.2	71.8	n.s.
Heptane, 3-ethyl-	286.5	241.7	242.4	35.7	n.s.
Nonane, 3,7	147.2	153.4	160.8	0.53	n.s.
Heptane, 2,2,4-trimethyl-	259.9	288.7	277.8	18.2	n.s.
Octane, 3,5-dimethyl-	225.4	247.9	279.6	28.1	n.s.
3-Methyl-3-hexene	123.6	122.8	125.7	1.47	n.s.
2-Octene, 4-ethyl-, (E)-	136.7	137.6	187.5	27.4	n.s.
Heptane, 3-ethyl-5-methylene-	274.2	282.3	274.7	19.3	n.s.
3-Ethyl-3-methylheptane	81.3	82.2	87.3	9.23	n.s.
Pentane, 3,3-dimethyl-	37.9	38.5	33.6	4.12	n.s.
Undecane, 6,6-dimethyl	97.6	88.1	96.7	12.3	n.s.
Nonane, 5-methylene-	98.5	89.2	87.6	13.2	n.s.
2-Nonene, 3-methyl, (E)-	247.1	253.3	268.5	26.3	n.s.
Heptane,2,2,4,6,6-pentamethyl	2227.7	2618.6	2539.8	211.8	n.s.
Decane	336.0	364.4	346.7	32.7	n.s.
(Z)-4-Methyl-2-hexene	127.2	173.2	147.8	23.6	n.s.
2,2,4,4-Tetramethyloctane	197.1	223.2	188.6	27.0	n.s.
Undecane, 5,5-dimethyl-	145.5	177.4	179.5	25.3	n.s.
Dodecane,2,6,10-trimethyl-	127.5	166.2	138.9	22.4	n.s.
Dodecane, 4-methyl-	88.2	108.8	99.6	14.2	n.s.
2-Decene, 3-methyl, (Z)-	52.3	51.8	59.6	8.10	n.s.
Undecane	1057.5	1205.4	1198.6	152.8	n.s.
2-Undecene, 9-methyl-, (Z)-	225.4	255.5	234.1	26.3	n.s.
4,4-Dipropilheptane	14.2	13.7	13.9	0.66	n.s.
Pentane, 3,3-diethyl-	43.8	44.6	45.4	1.24	n.s.
Dodecane, 2-methyl-6-propyl-	113.2	118.3	112.5	11.8	n.s.
2-Undecene, 3-methyl-, (E)-	38.2	35.4	42.2	2.15	n.s.
Dodecane	490.7	435.0	478.6	76.4	n.s.
Pentadecane, 6-methyl-	32.4	33.5	37.6	5.76	n.s.
Decane, 2,3,6-trimethyl	18.9	22.7	24.7	0.63	n.s.
Tridecane	99.9	115.2	118.2	10.7	n.s.
Tridecane, 3-methyl-	14.8	15.5	14.9	0.37	n.s.
Tetradecane	13.3	16.0	16.9	0.21	n.s.
**Total hydrocarbons**	**8961.5**	**10,987.6**	**9609.6**	**1024.9**	n.s.
Benzene	31.1 ^a^	125.4 ^b^	127.5 ^b^	12.2	*
Toluene	112.1	97.1	99.6	8.63	n.s.
Ethylbenzene	140.6 ^a^	393.4 ^b^	380.8 ^b^	48.5	*
Benzene, 1,3-dimethyl-	287.1	300.5	315.4	49.4	n.s.
p-Xylene	69.4	67.1	63.9	12.2	n.s.
3-Carene	94.1 ^a^	180.0 ^b^	185.2 ^b^	10.1	*
**Total aromatic hydrocarbons**	7**34.4 ^a^**	**1163.5 ^b^**	**1172.4 ^b^**	**141.0**	*

Significance: * (*p* < 0.05). n.s.: not significant. Different letters in the same row denote statistical difference: (^a,b^ *p* < 0.05).

**Table 5:** Effects of ageing period on donkey meat ketone and alcohol content. (AU × 10^3^/g).

Volatile Compound	2 Days	8 Days	15 Days	SEM	*p*-Value
2,3-Butanedione	237.5 ^a^	260.7 ^a^	302.1 ^b^	84.5	*
2-Butanone	198.3	211.4	185.6	28.3	n.s.
2-Pentanone	15.5	18.2	16.4	0.65	n.s.
3-Pentanone	79.4	105.5	88.7	22.1	n.s.
2,3-Pentanedione	84.6	81.2	100.2	17.4	n.s.
Acetoin	134.7	169.8	177.5	23.8	n.s.
3-Heptanone	15.1 ^a^	27.8 ^b^	30.1 ^b^	2.81	*
2-Heptanone	107.3 ^a^	145.5 ^a^	363.6 ^b^	80.2	*
4-Hexene-3-one,5-methyl-	21.5 ^a^	26.2 ^a^	38.5 ^b^	1.20	*
Butyrolactone	141.2	163.5	135.1	15.4	n.s.
2(5H)-Furanone	105.7	176.5	138.9	12.3	n.s.
3-Octen-2-one	26.1	28.2	27.3	1.21	n.s.
3-Octanone, 2-methyl-	28.5	30.2	29.8	1.02	n.s.
**Total ketones**	**1195.4 ^a^**	**1444.7 ^a^**	**1633.8 ^b^**	**291.2**	*
Cyclobutanol	158.1	177.2	185.0	14.5	n.s.
1-Pentanol	631.2 ^a^	652.4 ^a^	1.442.5 ^b^	231.5	*
1-Hexanol	2165.3 ^a^	1872.4 ^a^	3743.6 ^b^	102.5	*
1-Heptanol	80.5	95.1	100.2	27.2	n.s.
1-Octen-3-ol	182.5 ^a^	214.1 ^a^	388.9 ^b^	62.6	*
n-tridecan-1-ol	124.0	166.4	164.3	19.4	n.s.
1-Heptanol, 2,4-diethyl	165.4	188.5	194.1	20.1	n.s.
1-Decanol	19.2	20.8	22.1	1.26	n.s.
1-Tetradecanol	26.5	23.1	28.6	0.74	n.s.
1-Decanol, 2-hexyl-	18.8	18.2	18.1	1.11	n.s.
1-Butanol, 2-methyl-	14.1	13.8	13.3	0.55	n.s.
1-Octanol, 2-methyl-	17.7	13.5	16.9	1.01	n.s.
**Total alcohols**	**3603.3 ^A^**	**3455.5 ^A^**	**6317.6 ^B^**	**447.8**	**

Significance: * (*p* < 0.05); ** (*p* < 0.01). n.s.: not significant. Different letters in the same row denote statistical difference: (^a,b^ *p* < 0.05; ^A,B^ *p* < 0.01).

**Table 6:** Effects of ageing on donkey meat carboxylic acids, total nitrogen and sulphur compounds (AU × 10^3^/g).

Volatile Compound	1 Day	8 Days	15 Days	SEM	*p*-Value
Butanoic acid	93.6 ^a^	173.5 ^b^	177.8 ^b^	29.6	*
Hexanoic acid	71.7	62.3	81.6	17.5	n.s.
Formic acid	69.2	74.2	80.0	8.01	n.s.
**Total carboxylic acids**	**234.5 ^a^**	**310.0 ^b^**	**339.4 ^b^**	**45.1**	*
Diazene, dimethyl.	366.2	369.5	364.4	35.1	n.s.
Pyrazine, methyl-	158.2	92.1	89.5	11.1	*
2-Propen-1-amine	110.1	66.6	56.2	9.28	*
Pyrazine, 2,5-dimethyl-	265.3	254.7	251.4	16.2	n.s.
Pyrazine, trimethyl-	270.2 ^a^	152.4 ^b^	144.2 ^b^	21.3	*
**Total nitrogen compounds**	**1170.0 ^a^**	**935.3 ^b^**	**905.7 ^b^**	**89.9**	*
Dimethylsulphide	22.1	24.6	25.3	2.60	n.s.
Carbon disulphide	115.3	117.5	111.1	12.5	n.s.
**Total sulphur compounds**	**137.4**	**142.1**	**136.4**	**16.2**	**n.s.**

Significance: * (*p* < 0.05). n.s.: not significant. Different letters in the row denote statistical difference: (^a,b^ *p* < 0.05).
